# Practical Medicine

**Published:** 1878-11

**Authors:** 


### Practical Medicine.

On some forms of Chronic Catarrh of the Stomach. —Wm. B. Neftel. Part II. (*Medical Record*, Sept. 28, 1878.)

In their dietetic management a variety of wholesome food is required; including both farinaceous substances and animal food —especially meat, beef tea and soups. But beef tea, soups and meat juice contain only the aqueous extract of meat and leave earthy phosphates and albumen in the residuum and cannot entirely replace meat in substance.

Meat should not be allowed raw or underdone, from the danger of animal parasites. For the same reason drinking of fresh blood should be condemned.

Meat should be well and palatably prepared, not greasy nor burnt.

Poultry and game (well cooked) are generally allowed, although goose, duck, etc., are difficult to digest, and have to be used with discretion.

Next to meat, fish is very digestible and nourishing, but must be well cooked. That it does supply more phosphorus to the system than meat, milk, eggs, bread, etc., is an exaggeration.

Fresh milk (with alkaline reaction) is a most excellent article of diet for dyspeptics. Cream cannot be a substitute for milk, and is rather injurious to dyspeptics.

Eggs are the most nutritious dietetic article, and may be cooked hard or soft, though when not perfectly fresh they should be cooked thoroughly.

Bread is the representative of the nutritive vegetable substances. For dyspeptics it ought to contain no admixture of fat, milk, sugar, molasses, etc., but be made very light, simply with water, salt, and good yeast, and well baked. The crust of well baked bread increases its digestible properties.

Peas, beans and other leguminosa are very nutritious, but in cooking them for dyspeptics their capsules should be eliminated as they cause flatulence.

Potatoes have less nutritive value, though rich in starch, potassium and phosphoric acid, yet contain 70 to 80 per cent. of water. Dyspeptics must forego eating too many potatoes.

Arrowroot, sago, tapioca, are identical with the starch of potatoes, and can be used with benefit.

Fresh fruits contain over 70 per cent. of water, and do not belong to a nourishing diet—but are reckoned among the *refreshing* dietetic substances. Lemon juice contains a large amount of potassic salts. In chronic gastric catarrh, however, fresh fruits are not well digested.

Vegetables (such as beets, cabbage, lettuce, etc.,) are not easily digested by dyspeptics, and may cause pain; they should be avoided.

Tea and coffee and even chocolate, if it agrees, may be allowed. Although often prohibited, they are very wholesome and useful beverages. "They all contain the same active principle, a *nitrogenous* organic basis—thein, caffein theobromin, which are chemically and physiologically identical, and which act upon the system in the same way as the extractive substance of meat."

"The infusion of tea contains, besides, a considerable amount of the salts of iron and manganese. Their ash is similar to that of the blood. These beverages stimulate in a moderate degree the muscular and nervous systems, improve the circulation, and thus increase our physical and mental productivity."

If indulged in to excess, they diminish the appetite for that amount of food necessary for the healthy condition of the system.

Convalescents from chronic gastric catarrh, often inquire if they should restrain their appetite after complete recovery. The doctor thinks of the two evils—too much or too little food—the latter is the most objectionable, as it leads to a deficiency in the nutrition of some organ or of the whole system.

ANTISEPTIC INHALATION IN PHTHISIS. Eade. (*L'Union Médicale*, September 5, 1878, page 364.)

The author has recourse to carbolic acid vapors to diminish the muco-purulent secretion, and to lessen the cough and debility incident thereto in consumptives. Pour into a narrow-necked jug or pitcher, 250 grams of moderately warm water, and add 60 centigrams of carbolic acid. Shake and let the patient inhale the vapors for ten minutes. This may be done three to five times in the twenty-four hours, during the night if necessary. It is also useful in bronchitis to facilitate expectoration.

Advantages of a Single Puncture of Each Arm in the Vaccination of Very Young Children.—(*Dr. Hugues, Gazette Obstétricale from Nice Médicale, Sept.* 5, 1878, *page* 259.

The author establishes the two following conclusions:

1st. Contrary to the general opinion, it must not be believed that variola being rare in the first two or three months of life, it is not necessary to vaccinate at this age. He has observed several cases of small-pox in children less than three months old, which he had refused to vaccinate. He therefore vaccinates any child now at the slightest wish of the parents. But having formerly made three punctures in each arm, and seen several grave accidents due to extent or intensity of inflammation, he was led to inquire if a single puncture would not be sufficient.

2d. It is entirely sufficient to make but a single puncture in each arm in very young children. In eleven children re-vaccinated at later periods, and even with three punctures, not one was susceptible to the influence of the virus.

